# Glacial History of a Modern Invader: Phylogeography and Species Distribution Modelling of the Asian Tiger Mosquito *Aedes albopictus*

**DOI:** 10.1371/journal.pone.0044515

**Published:** 2012-09-06

**Authors:** Daniele Porretta, Valentina Mastrantonio, Romeo Bellini, Pradya Somboon, Sandra Urbanelli

**Affiliations:** 1 Department of Ecological and Biological Sciences, Tuscia University, Viterbo, Italy; 2 Department of Environmental Biology, University “La Sapienza” of Rome, Rome, Italy; 3 Agriculture and Environment Centre “G. Nicoli”, Crevalcore, Italy; 4 Department of Parasitology, Chiang Mai University, Chiang Mai, Thailand; Centro de Pesquisas René Rachou, Brazil

## Abstract

**Background:**

The tiger mosquito, *Aedes albopictus*, is one of the 100 most invasive species in the world and a vector of human diseases. In the last 30 years, it has spread from its native range in East Asia to Africa, Europe, and the Americas. Although this modern invasion has been the focus of many studies, the history of the species’ native populations remains poorly understood. Here, we aimed to assess the role of Pleistocene climatic changes in shaping the current distribution of the species in its native range.

**Methodology/Principal Findings:**

We investigated the phylogeography, historical demography, and species distribution of *Ae. albopictus* native populations at the Last Glacial Maximum (LGM). Individuals from 16 localities from East Asia were analyzed for sequence variation at two mitochondrial genes. No phylogeographic structure was observed across the study area. Demographic analyses showed a signature of population expansion that started roughly 70,000 years BP. The occurrence of a continuous and climatically suitable area comprising Southeast China, Indochinese Peninsula, and Sundaland during LGM was indicated by species distribution modelling.

**Conclusions/Significance:**

Our results suggest an evolutionary scenario in which, during the last glacial phase, *Ae. albopictus* did not experience a fragmentation phase but rather persisted in interconnected populations and experienced demographic growth. The wide ecological flexibility of the species probably played a crucial role in its response to glacial-induced environmental changes. Currently, there is little information on the impact of Pleistocene climatic changes on animal species in East Asia. Most of the studies focused on forest-associated species and suggested cycles of glacial fragmentation and post-glacial expansion. The case of *Ae. albopictus*, which exhibits a pattern not previously observed in the study area, adds an important piece to our understanding of the Pleistocene history of East Asian biota.

## Introduction

The Pleistocene was a time of intensive climatic fluctuations, during which glacial–interglacial cycles occurred at irregular intervals with varying durations [1]. These climatic changes substantially influenced both the current distribution of species and their genetic diversity [1]–[5]. An understanding of how species responded to past environmental change has important implications for long-term conservation of biodiversity and could also be useful for understanding species’ responses to ongoing climatic change [2], [3], [6]–[10]. Understanding these responses may be of particular interest in the case of disease vectors and/or invasive species [11]–[13]. In recent decades, much light has been shed on the general features of how species coped with Pleistocene climatic changes [3], [4], [14]–[16]. However, our knowledge is uneven across the globe in consequence of the disparity in the numbers of studies conducted in different regions. The impact of these climatic changes has been well-documented for temperate taxa that inhabit North America and Europe, whereas it has received little attention for species of Asian regions [3], [17].

In Far East Asian regions, Pleistocene climatic changes caused dramatic environmental changes, although the glacial advances were not as extensive as those in Europe or the Americas [18]. During the Last Glacial Maximum (LGM; ∼21,000 years BP), at mid-latitude (30–40° N), steppe and desert biomes extended southward as far as the modern coast of Eastern China. The broadleaf evergreen warm mixed forest shifted southward into the lowlands as far as 24° N, and tropical forests were restricted to the northern part of the Indochinese Peninsula [18], [19]. Open habitats such as savannah extended from the Malay Peninsula southward [20].

Most phylogeographic studies of animal species in these regions have focused on forest-associated species to investigate whether and how they were affected by ice-age-driven changes in forest distribution, both at mid-latitude and in the Indochinese Peninsula [21]–[26]. Attempts to identify common patterns are being carried out for those areas where a greater number of studies have been conducted [23], [25], [26]. For instance, it has been suggested that in the Indochinese Peninsula, mosquito species associated with tropical forests experienced fragmentation phases into separate northeastern and northwestern refugia. Subsequent post-glacial expansion southward from glacial refugia has also been suggested, leading to the formation of a suture zone between intraspecific genetic lineages along the Thailand–Myanmar border [26].

Despite their abundance, animal species not strictly associated with forest habitats have been little studied; as a result, how they coped with Pleistocene climatic changes remains overlooked. To contribute towards filling this gap, we investigated the Pleistocene evolutionary history of the Asian tiger mosquito *Aedes albopictus*, a generalist species with wide ecological plasticity. It is adapted to both tropical and temperate zones and is distributed across East Asia, from Indonesia to Korea on the north–south axis and from Japan to India on the east–west axis. In temperate regions, *Ae. albopictus* overwinters by photoperiodic egg diapause ([27], [28] and references therein, [29]). It is common in forested areas as well as open habitats, in both suburban and rural areas. The two most typical microhabitats of *Ae. albopictus* appear to be tree holes and man-made containers of any material, from glass to stone, plastic, wood, or rubber [27]. The females need very little water for oviposition and feed on a wide range of vertebrate hosts [27], [30]. Evidence of the species’ extraordinary ecological flexibility and its ability to exploit different habitats comes from its recent history. *Ae. albopictus* is an invasive species that, in the last few decades, has spread from Asia to the Americas, Europe, Africa, and Australia, as a result of human activities [31]. During its worldwide spread, the species has successfully competed with co-occurring mosquito species and has adapted to a variety of different environmental conditions [28], [32]–[34].

Owing to its wide ecological plasticity, *Ae. albopictus* may not conform to the scenario of past fragmentation during glacial phases that has been suggested for other species [35]–[39]. Previous genetic studies on *Ae. albopictus* populations by using nuclear markers have shown a lack of genetic discontinuity across most of the geographic range of the species (pairwise Nei’s unbiased genetic distance [40] never higher than 0.08), which could support this hypothesis [41], [42]. Here, we used two sets of data to investigate how the species coped with Pleistocene climatic changes. First, we used sequence variation data for two mitochondrial DNA genes to investigate the phylogeography and historical demography of the species across East Asia. Second, we used species distribution modelling (SDM) to reconstruct the species distribution during LGM.

The utility of integrating SDM into phylogeography has recently been highlighted by several researchers, who have shown that the inferences from one approach can be investigated and even potentially corroborated by another [15], [43]–[47]. Under a scenario of prolonged allopatric divergence in multiple refugia across East Asia during the glacial phase, we would expect to observe groups of genetically distinct populations, or highly diverging reciprocally monophyletic lineages or secondary contact zones resulting from admixture between populations previously restricted in separate refugia [2], [3]. Under this scenario, SDM would also show the occurrence of multiple geographically separated areas of suitable climatic conditions. Under an alternative scenario of a lack of population fragmentation during this period, we would expect a lack of the above genetic signatures, and SDM would show the occurrence of continuous suitable climatic conditions across the study area.

## Materials and Methods

### Ethics Statements

No specific permits were required for the described field studies. The localities, where the mosquitoes were collected, are no private or protected areas, and the study did not involve endangered or protected species.

### Genetic Analyses

**Figure 1:**
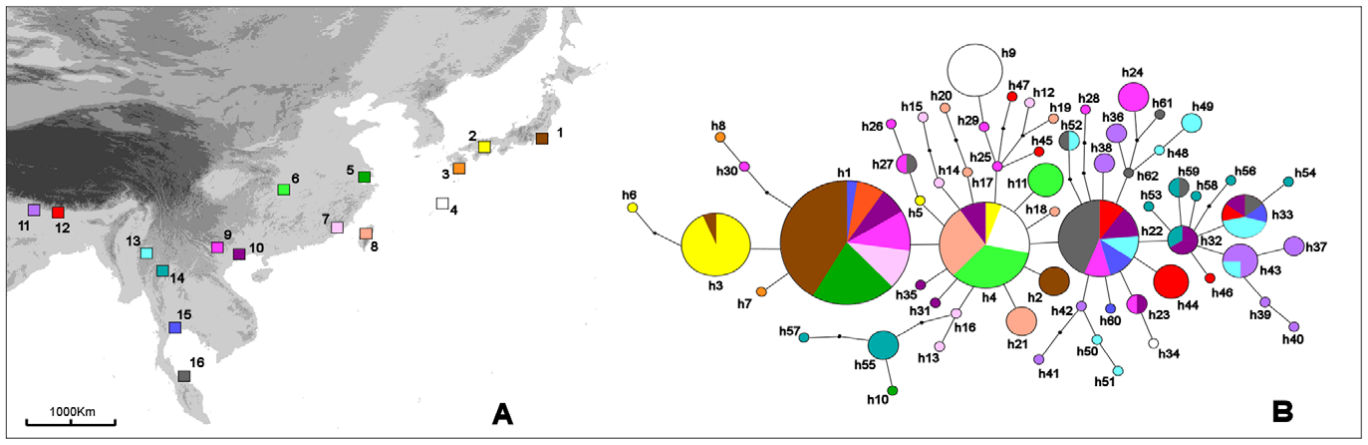
Sampling sites and phylogenetic relationships among mitochondrial haplotypes. (A) Map of East Asia showing the sampling sites of *Aedes albopictus*. For localities details see Table 1. (B) Statistical parsimony network, constructed using TCS software, showing phylogenetic relationships among the haplotypes found. Haplotypes are shown as circles with sizes corresponding to their frequencies in the total sample and colour corresponding to populations where they have been observed. Haplotypes are encoded as in Table 1. Dots indicate missing intermediate haplotypes.

Adults of *Ae. albopictus* were collected from 16 localities through the East Asia to cover the range of distribution of the species in both tropical and temperate areas (Figure 1A, Table 1) and the refugia regions inferred for other animal species [21]–[26]. By using aspirators, mosquitoes resting near humans outdoors were captured in three-five places per site sampling. Then, in laboratory, mosquitoes were killed by exposure to ethyl acetate vapour, and were placed individually in eppendorf 1.5 ml tube with ethanol 80%.

**Table 1:** Geographical origin, sample size and haplotypes’ distribution for the 16 sampled populations of *Aedes albopictus* (haplotypes in bold have been found in two or more localities; the number in brackets indicates how many time a haplotype has been observed at a particular locality).

Code	Locality (Country)	Lat.	Long.	Sample size	Haplotypes	*h*	*π*
1.	Choral uji (Japan)	34°21′ N	133°29′ E	14	**h1**(10), h2(3), **h3**	0.472±0.136	0.0006±0.0005
2.	Tanega Shima (Japan)	30°36′ N	130°56′ E	15	**h3**(12), **h4**, h5, h6,	0.371±0.153	0.0006±0.0005
3.	Nagasaki (Japan)	32°47′ N	129°49′ E	4	**h1**(2), h7, h8	–	–
4.	Okinawa (Japan	26°20′ N	127°52′ E	11	**h4**(4), h9(7)	0.509±0.100	0.0010±0.0008
5.	Hangzhou (China)	30°16′ N	120°11′ E	7	**h1**(6), h10	0.286±0.200	0.0006±0.0005
6.	Zhenyuan (China)	27°13′ N	108°18′ E	10	**h4**(6), h11(4)	0.533±0.095	0.0004±0.0004
7.	Shantou (China)	23°22′ N	116°40′ E	8	**h1**(3), h12, h13, h14, h15, h16	0.893±0.111	0.0019±0.0013
8.	Taoyuan (Taiwan, China)	23°12′ N	120°50′ E	11	**h4**(5), h17, h18, h19, h20, h21(2)	0.800±0.114	0.0012±0.0008
9.	Vinh Phuc (Vietnam)	21°18′ N	105°34′ E	15	**h1**(3), **h22**(2), **h23**, h24(3), h25,h26, **h27**, h28, h29, h30	0.933±0.050	0.0023±0.0014
10.	Hanoi (Vietnam)	20°55′ N	105°51′ E	13	**h1**(2), **h4**(2), **h22**(2), **h23**, h31,**h32**(2), **h33**, **h34**, h35	0.950±0.042	0.0014±0.0010
11.	Phuntsholing (Bhutan)	26°34′ N	89°16′ E	11	h36(2), h37(2), h38(2), h39, h40,h41, h42, **h43**	0.946±0.053	0.0021±0.0014
12.	Gelephu (Bhutan)	26°36′ N	90°31′ E	10	**h22**(2), **h33**, h44(4), h45, h46, h47	0.844±0.103	0.0018±0.0012
13.	Chiang Mai (Thailand)	18°51′ N	98°36′ E	13	**h22**(2), **h33**(2), **h43**(3), h48,h49(2), h50, h51, **h52**	0.923±0.050	0.0022±0.0013
14.	Lampang (Thailand)	18°41′ N	99°47′ E	10	**h32**, h53, h54, h55(3), h56, h57,h58, **h59**	0.933±0.077	0.0022±0.0014
15.	Ratchaburi (Thailand)	13°10′ N	99°50′ E	6	**h1**, **h22**(2), **h33**, **h34**, h60	0.933±0.122	0.0014±0.0010
16.	Songkhla (Thailand)	6°48′ N	100°35′ E	16	**h22**(9), **h27**, **h33**, **h34**, **h52**, **h59**,h61, h62	0.700±0.127	0.0017±0.0021

Haplotype diversity, *h* (± SD), and nucleotidic diversity, *π* (± SD), are also given.

Total genomic DNA was extracted from single mosquitoes following the standard CTAB (cetyltrimethyl ammonium bromide) protocol [48]. Partial sequences of the mitochondrial DNA genes encoding for the cytochrome oxidase I (*COI*) and nicotinamide adenine dinucleotide dehydrogenase subunit 5 (*ND5*) were obtained through Polymerase Chain Reaction **(**PCR). The primers TW-J-1305 and TK-N-3782 [38] were used to amplify and sequence ∼2000 base pairs including the regions *COI*, *tRNA-Leu* and *COII*. The following primers were then designed to amplify and sequencing two regions of the *COI* gene: *Aeal*COIa-f 5′- aaaaagatgtatttaaatttcggtctg-3′ and *Aeal*COIa-r 5′-tgtaattgttactgctcatgctttt-3′; *Aeal*COIb-f 5′-ttattacacaagaaagaggaaaaa-3′ and *Aeal*COIb-r 5′-cattgcactaatctgccata- 3′. The *ND5* gene region was amplified using the primers designed by Birungi & Munstermann [49]. Each PCR amplification was performed in 25 µl including 1x Buffer, 200 µM dNTPs, 2.5 mM MgCl_2_, primers at 0.2 µM, 0.5 units of Taq DNA Polymerase (GoTaq™ DNA Polymerase, Promega), and 20 ng of genomic DNA extracted from single mosquito. The PCR cycling procedure was: 95°C for 5 min followed by 34 cycles at 93°C for 1 min, annealing temperature: 55°C for *COI* and *COIb*; 50°C for *ND5* for 1 min, 72°C for 1 min 30s, and a single final step at 72°C for 10 min. PCR sequences were obtained using an ABI PRISM 3700 DNA sequencer by Macrogen Inc. (www.macrogen.com). All individuals analysed were sequenced using both forward and reverse primers, and all haplotypes that were found unique (see Result section) were re-amplified by using high fidelity Taq DNA Polymerase (Phusion® High-Fidelity DNA Polymerase, Fermentas) and re-sequenced to check for consistency.

Sequences were edited and aligned using the software Chromas 2.33 (Technelysium Pty Ltd, Australia) and Clustalx 2.0 [50] respectively.

Nucleotide and amino-acidic polymorphisms of the *COI* and *ND5* genes were assessed using the software Mega 5.0 [51]. For all the subsequent analyses the two genes were concatenated since the partition-homogeneity test – conducted with 1000 replicates using the software Paup* 4.0b10 [52] - did not reject the null hypothesis of homogeneity of their phylogenetic signals. The software jModelTest version 0.1.1 [53] was used to find the optimal model of sequence evolution for our data using the Akaike Information Criterion. The HKY+I+Γ substitution model with the alpha value of the gamma distribution = 0.812 and the proportion of invariable sites I = 0.862 was shown as the best-fit model for the data. Haplotype diversity (*h*) and nucleotide diversity (*π*) were estimated for each locality (with the exception of Nagasaki population where 4 individuals were sampled) and for the overall dataset, using the software Arlequin 3.1 [54].

The genealogical relationships between haplotypes were investigated by constructing a phylogenetic network [55]. We used the statistical parsimony algorithm described by Templeton et al. [56] and implemented in the TCS software [56], applying a 95% cutoff for the probability of a parsimonious connection. To check for consistency [57] we also used the median-joining (MJ) network algorithm as implemented in the Network 4.6.1.0 software (Fluxus Technology Ltd). The loops in the resulting phylogenetic network were resolved by applying the criteria described by Pfenninger & Posada [58].

The possible occurrence of groups of genetically distinct populations was investigated by Spatial Analysis of Molecular Variance as implemented in the software samova 1.0 [59]. This method defines groups of populations on the basis of genetic data, without any *a priori* hypothesis of the expected structure. Given a user-defined number K of groups of populations, samova uses a simulated annealing procedure to define groups of populations that are as genetically homogenous as possible (among population differentiation index, *F_SC_,* minimized) and maximally differentiated from one another (among groups differentiation index, *F_CT_,* maximized). We tested K values ranging from 2 to 15 and, to check for consistency, we conducted each analysis five times, with 10,000 independent annealing processes each.

To investigate signatures of past demographic changes we analysed the distribution of pairwise nucleotide differences between haplotypes (mismatch distribution) as implemented in the software Arlequin 3.1. In populations that have undergone a sudden demographic expansion, the mismatch distributions are expected to be smooth and bell-shaped [60]. The sum of square deviations (SSD) between estimated and observed mismatch distributions was used as goodness-of-fit statistics and its significance was tested through 1000 replicates. To further detect signatures of past demographic changes, we also used Fu’s *F_S_* neutrality test [61]. Large negative values of the parameter *F_S_* indicate an excess of singleton mutations, which is expected both under departures from selective neutrality and in populations which have experienced a recent demographic expansion. It was computed on Arlequin 3.1, and its significance was assessed through 1000 replicates. Finally, historical demographic trend was investigated using the Bayesian skyline plot (BSP) method by Drummond et al. [62], as implemented in the software beast 1.6.1 [63]. This method estimates the posterior distribution of the effective population size through time using a Markov chain Monte Carlo (MCMC) sampling procedure. We used the HKY+I+Γ substitution model of sequence evolution. To give an approximate time scale to the inferred demographic trend, we used a mutation rate of 0.0115 substitutions/site/lineage/Myr, based on the divergence rate of 2.3% per million years [64]. It may be considered as an appropriate rate for mosquitoes, as this divergence rate has been estimated from several arthropod datasets, including several Diptera. Furthermore, it has been recently used in different mosquito species and the demographic trend inferred was shown to accord with independent paleoenvironmental data [26]. A relaxed molecular clock model was used, with uncorrelated rates drawn from a log-normal distribution. Preliminary batch runs were carried out using the auto-optimization procedure implemented in beast in order to fine-tune MCMC parameters. The MCMCs were finally run for 20 million steps, and sampled every 1000 steps. The first two million steps were discarded as burn-in, after checking the likelihood history with tracer 1.4 [65]. The same software was used to analyse the results of the BSP analyses. Five replicates of the BSP analysis were run to check for consistency among different runs.

### Species Distribution Modelling

The Species Distribution Model (SDM) for *Ae. albopictus* was developed using the “maximum entropy model” as implemented in Maxent 3.3.3e [66]. This software reconstructs the potential distribution of a species combining presence-only data with climatic layers, distinguishing presence from random [66]. Although our study is focused on East Asian regions, all SDMs were constructed including also the Indonesian Islands since, during the LGM, they were connected to the mainland Asia as a consequence of the Sundaland exposure (see Discussion). All models were developed using a total of 153 occurrence points of *Ae. albopictus*, including records from literature, records from the “Global Biodiversity Information Facility” (GBIF) online database (http://data.gbif.org) and our sampled localities. We selected all georeferenced localities, while among those without geographical coordinates, we used only localities for which exhaustive geographical data are furnished by the authors. These localities were then georeferenced using the software Google Earth.

The SDM was developed for Current, Last Glacial Maximum (LGM) and Last Inter-Glacial (LIG) conditions. To reconstruct the ecological niche of LGM conditions, we used two general circulation models (GCM): the Community Climate System Model 3 (CCSM 3) [67] and the Model for Interdisciplinary Research on Climate (MIROC 3.2) [68]. For all models, we used the bioclimatic variables available in WorldClim database (http://www.worldclim.org/). These 19 variables, derived from monthly temperature and rainfall values, were downloaded at spatial resolution of 2.5 arc-minutes for current and LGM conditions, and at spatial resolution of 30 arc-seconds for LIG conditions.

Pearson’s correlation matrix was generated for all 19 variables to quantify the correlation between them. For pairs that were highly correlated (correlation coefficient ≥0.75) we chose the variable more biologically meaningful for the species [27]. Finally, all SDMs were constructed using four variables, including: BIO6 (Min Temperature in Coldest Period); BIO8 (Mean Temperature of Wettest Quarter); BIO18 (Precipitation of Warmest quarter) and BIO19 (Precipitation of Coldest Quarter).

In Maxent, each run was conducted using the default convergence threshold (10^−5^) and maximum number of iterations (500) values. For the regularization parameter *β*, as suggested by Warren & Seifert [69], we tested the values 1, 3, 5, 7, 9, 11, 13, 15, 17 and 19 using the software EnmTools [70]. The best model, chosen using the corrected Akaike Information Criterion (AIC_c_), was one for which *β* = 3. Twenty-five percent of record points were used for model testing, and the final SDMs performance was evaluated using the area under receiver operating characteristic (ROC) curve (AUC). An AUC score above 0.7 is considered good model performance [71]. All SDM predictions were visualized in DIVA GIS 5.2 [72]. To provide a consensus map of the species distribution at LGM we combined the CCSM and MIROC predictions (available under request to the authors) by including areas predicted as suitable by both models, while discarding areas where no agreement occurred [15]. We used the software ImageJ [73] to compute the area of suitable habitats of the species under the LIG and LGM conditions using the “minimum training presence” threshold for presence-absence.

## Results

### Genetic Data

A final sequence alignment including 1458 nucleotides (364 from the *ND5* gene fragment, 405 and 689 base pairs respectively for *Aeal*COIa and *Aeal*COIb primer pairs) was obtained for 174 individuals. Sixty two haplotypes were found (GenBank Accession numbers: JQ436947-JQ437008) defined by 54 nucleotide substitutions (32 parsimony informative). Forty one nucleotide substitutions were located at the third codon position, five at the second codon position and eight at the first one. Thirteen non-synonymous nucleotide substitutions were found. Overall haplotype and nucleotide diversity estimates were 0.945 (±0.009) and 0.0021 (±0.0012), respectively.

No apparent association between haplotypes and geography was observed. The three more frequent and centrally placed haplotypes in the genealogical network (h1, h4, and h22), were found in populations spanning the entire east-west and north-south extension of the study area (Table 1, Figure 1).

Phylogenetic relationships among the 62 haplotypes found are shown in Figure 1B. Both statistical parsimony and median-joining networks showed the same topology, so only the statistical parsimony network is shown. The uncorrected mean sequence divergence among haplotypes was 0.30%. A large portion (39/62, 63%) of the haplotypes found were unique (see also Table 1). No haplotype-groups were apparent being all haplotypes connected to each other by no more than 3 nucleotide substitutions (Figure 1B).

By the spatial analysis of molecular variance (SAMOVA) no groups of genetically distinct populations were evidenced. All partitions of the sampling areas for each *K* value showed no significant increment in the *F_CT_* values. They showed a very narrow range (12/14 values were between 0.28 and 0.36) and the highest value (*F_CT_* = 0.40) was observed for *K* = 15, when all populations minus one were separated (Figure S1).

The haplotype diversity and nucleotide diversity estimates for each population are shown in Table 1. They ranged from 0.286 (sample 5, China) to 0.950 (sample 10, Vietnam) and from 0.0004 (sample 6, China) to 0.0023 (sample 9, Vietnam). The geographical distribution of haplotypes and the indices of population diversity showed the occurrence of an uneven distribution of genetic diversity throughout the study area (Table 1, Figure 1). The populations from Bhutan, Southern China, and Indochinese Peninsula showed the highest haplotype diversity. In these samples we also found all the most common haplotypes (h1, h4, h22) and those centrally placed in the network (h1, h4, h22, but also h25, h32).

**Figure 2:**
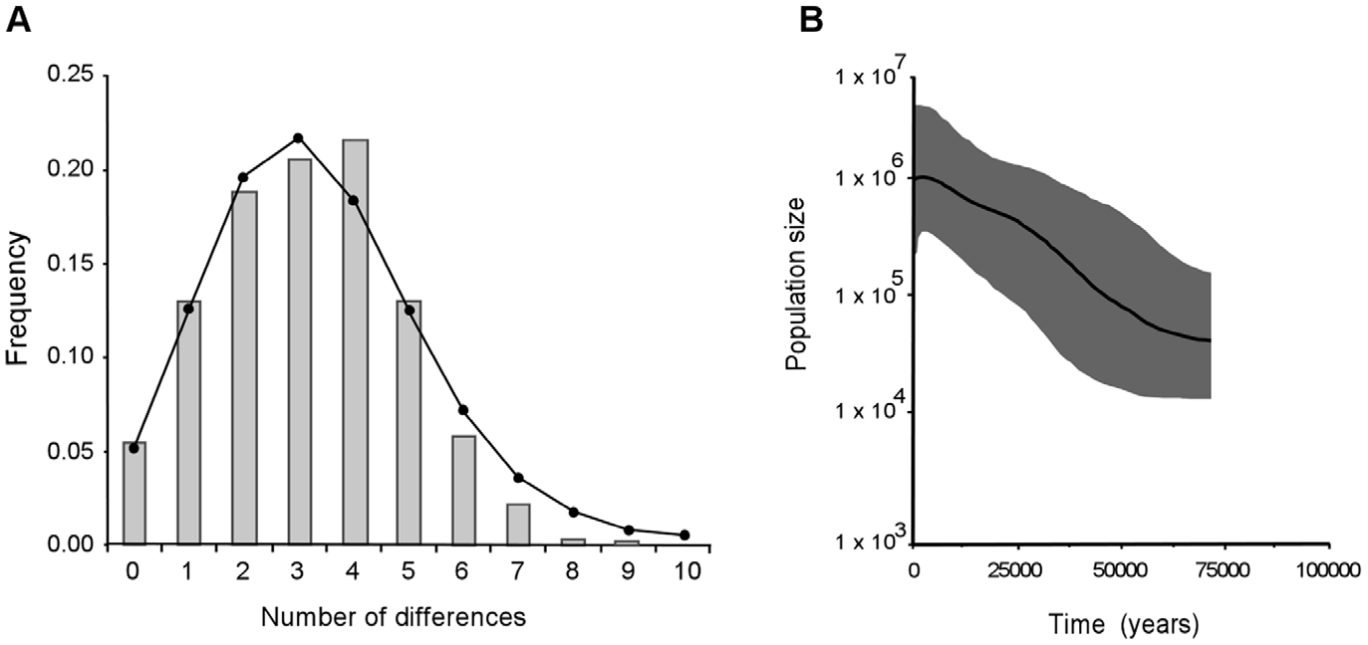
Historical demographic analyses. (A) Mismatch distribution computed using the software Arlequin 3.1. Histograms show the observed distribution; lines show the expected distribution under a model of sudden population expansion. (B) Bayesian Skyline Plot, constructed using the software beast 1.6.1. Population size (*y*-axis) is measured as the product of effective population size per generation length (*N*_e_τ). The solid line is the median estimate, and the grey areas show the 95% Highest Posterior Density (HPD) limits. Time (***x*** axis) is expressed in years before present (BP).

Demographic analyses showed the occurrence of past demographic expansion using both separated (data not shown) and concatenated *COI* and *ND5* genes (Figure 2). The mismatch distributions appeared smooth and bell-shaped. The SSD statistic supported a close fit of the observed distribution to the distribution expected under population expansion model (SSD = 0.002, *P* = 0.46) (Figure 2A). The past occurrence of a demographic expansion was also suggested by Fu’s *F*_s_ and the Bayesian skyline plot method (BSP). The former was large, negative and highly significant (*F*_s_ = −26.244, *P*<0.001), showing the occurrence of a larger than expected number of singleton mutations, as expected for populations that underwent a population expansion. The demographic trend inferred by the BSP also indicated the advent of a phase of demographic growth, which roughly started about 70,000 years BP until recently (Figure 2B).

### Species Distribution Modelling

**Figure 3:**
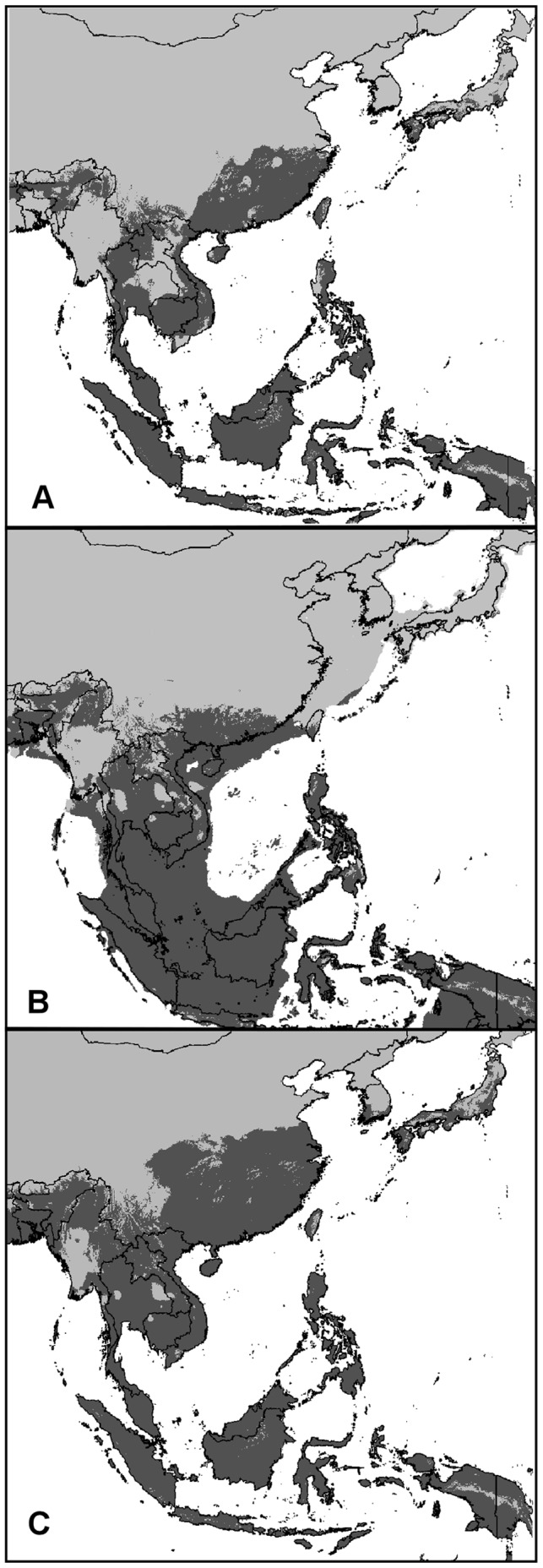
Bioclimatic models for *Aedes albopictus.* (A) Last Interglacial (LIG; ∼140,000–120,000 years BP); (B) Last Glacial Maximum (LGM, ∼21,000 years BP); (C) current conditions. All models were developed using the “maximum entropy model” as implemented in the software Maxent 3.3.3e. In dark grey are shown the areas predicted as suitable by both the CCSM and MIROC models.

The Maxent models predicted under LIG, LGM and current conditions of *Ae. albopictus* are shown on Figure 3. For the current condition model, the AUC for the training points was 0.935 and for test points was 0.899, indicating a good performance of the trained model. Our reconstruction under current conditions is concordant with those previously published by Benedict et al. [31] and Medley [34], which further supports the reliability of our predictions.

The distribution inferred at LIG shows the occurrence of climatically suitable areas both in tropical and subtropical regions (i.e. Indonesia, Indochinese Peninsula) and with lesser degree in temperate regions (i.e. China and Japan), similarly to the current conditions (Figure 3A and C). The projection at the LGM predicted the occurrence of mostly continuous areas with climatically suitable conditions for the species comprising South-East China, Indochinese peninsula and the emerged Sundaland. The emerging of the Sunda shelf resulted at the LGM in an increase of the potential suitable area for *Ae. albopictus* with respect to the LIG of 42% (by using the minimum training presence threshold, 0.092). At the higher latitudes, in the northernmost part of its current range of distribution (Northern China and Japan), areas of predicted suitable habitats were mainly absent (Figure 3B).

## Discussion

### mtDNA Diversity in Native Populations of *Aedes albopictus*

Previous studies of *Ae. albopictus* mitochondrial diversity have shown that native populations of this species harbour little or no mitochondrial genetic diversity ([49], [74] and references therein). In particular, an analysis by Birungi & Munstermann of 405 base pairs of the *ND5* mtDNA gene in samples derived from laboratory colonies revealed only 1 haplotype across an area spanning Japan, Malaysia, and Indonesia [49]. Armbruster et al. [74] suggested that the overall low mtDNA diversity observed with respect to nuclear markers in the native Asian populations was a result of a cytoplasmic ‘sweep’ caused by double infection of *Ae. albopictus* by the endosymbiont *Wolbachia.* An alternative explanation is that the mtDNA diversity of natural populations has been obscured by sampling bias: the *Ae. albopictus* individuals that were analysed were derived from laboratory colonies (F_10_–F_23_ generations) that did not represent the genetic diversity in natural populations. This explanation was supported by subsequent studies in which individuals from natural populations were used [75]–[79]. Our study lends further support to the hypothesis that the mtDNA diversity of natural populations has been overlooked. All the specimens used in this study showed positive results for infection with both *w*AlbA and *w*AlbB strains of *Wolbachia* ([80] and our unpublished data), as expected for *Ae. albopictus* populations [74]. We found not only a non-negligible genetic diversity–within the range of that observed in other mosquito species uninfected by *Wolbachia* [26], [48], [81]–but also an overall concordance between the genetic patterns observed at nuclear and mitochondrial markers; this would not be expected if mtDNA selective sweep had occurred [82]. Previous population genetics studies conducted using allozymes showed significant genetic differentiation between populations at a regional level, but a lack of discontinuity in the geographic distribution of genetic diversity at the level of mainland Asia as a whole [41], [42], [83]. Finally, the topology of the network showing the phylogenetic relationships between the mtDNA haplotypes (i.e., multiple starlike topology, Figure 1B) is quite different from that of the single star-like network expected under a scenario of recent selective sweep driven by maternally inherited symbionts such as *Wolbachia* [82]. Mitochondrial DNA has been widely used to infer the geographic origin of invasive populations of *Ae. albopictus* [49], [75], [77], [79]. The comparison between native and new colonizing populations is outside the scope of our study. However, our mtDNA survey adds new haplotypes that will supplement the poor genetic pool available to date and can be used in future studies.

### Late Pleistocene History of *Aedes albopictus*

The genetic pattern observed in *Ae. albopictus* populations is hard to reconcile with a scenario of fragmentation in multiple refugia across East Asia during the last glacial phase, as has been proposed for forest-associated species. Indeed, we found neither phylogeographic structure nor highly diverging reciprocally monophyletic lineages, as would be expected if a prolonged allopatric divergence had occurred. It might be possible to explain the lack of phylogeographic structure by admixture between populations previously restricted in separate refugia. However, in this case, we would expect the existence of a genetic signature of prior allopatric fragmentation (i.e., the occurrence of diverging monophyletic lineages or secondary contact zones), which we did not observe.

In contrast, the genetic pattern observed best fits with a scenario in which the species never experienced a prolonged allopatric divergence but rather persisted in a wide area, most likely across Southeast China and the Indochinese Peninsula. In these regions, we found not only a lack of phylogeographic structure but also higher levels of genetic diversity (Figure 1), as well as the occurrence of the most common haplotypes and those centrally placed in the network (the signature of areas of the refugia) [14].

This scenario is also consistent with the reconstruction of the species distribution at the LGM (Figure 3B). Although we used a conservative criterion to combine the CCSM and MIROC predictions, the models predicted the occurrence of continuous climatically suitable areas not only in the southernmost regions of Sundaland but also through most of the Indochinese Peninsula northward as far as Southeast China and Bhutan (Figure 3B). Here, the occurrence of interconnected areas with suitable climatic conditions for the species was inferred. It is notable that paleoenvironmental reconstructions also showed the occurrence of different habitats (such as forests or open habitats) in these regions [18] that *Ae. albopictus* could have exploited and maintained large interconnected populations.

In the genetic pattern observed, we also found the signature for past demographic changes in *Ae. albopictus* populations. The demographic trend inferred from the BSP is that of a gradual expansion starting about 70,000 years BP, that is, during the last glacial phase including the LGM. Although the lack of a calibrated molecular clock demands caution, paleoenvironmental reconstructions of the region and SDM at the LGM support the hypothesis that populations of the species expanded during this period.

During the last glacial phase in Southeast Asian regions, the glacio-eustatic lowering of the sea level fully exposed the Sunda shelf (Sundaland), which connected the major islands of Sumatra, Java, and Borneo to mainland Asia by land bridges [20], [84]. At 70,000 years BP, the sea level was 20–50 m below its present level, and all these islands were joined to mainland Asia [85]. As shown by the SDM, the emergence of Sundaland led to a non-negligible increase (42%) in climatically suitable areas for the species compared to the LIG (Figure 3A–B). These emerged areas, according to paleoenvironmental reconstructions, were covered by tropical forests in areas of Sumatra, Java, and Eastern Borneo and by open habitats, such as savannah, from the Malay Peninsula southward [84]. The availability of large amounts of suitable habitat and the occurrence of suitable climatic conditions in the emerged Sundaland (Figure 3B) could have had a positive influence on *Ae. albopictus* populations and could account for the expansion inferred from genetic data. The effect of glacial-induced lowland emergence on population dynamics has recently been investigated in the Sardo-Corso region in a study by Bisconti et al. [86], which showed that the emergence of lowland plains during the last glacial phase counterbalanced the negative demographic consequences of climatic changes during the last glacial phase by providing new suitable habitats and led to net demographic expansions in a temperate and thermophilic species, the tree frog *Hyla sarda*.

The inferred demographic expansion of *Ae. albopictus* populations also encompasses the post-glacial phase (from 14,000 years BP until recently, Figure 2B). Following the LGM, the amelioration of climatic conditions at mid-latitude could have sustained the demographic expansion of the species into the northernmost areas of its current distribution (Central and Northern China and Japan) (see also Figure 3). The signature of this colonization event is still detectable in the geographic pattern of genetic diversity of the species: the northernmost populations (sample 1–6, Table 1) show lower levels of genetic diversity and a higher percentage of derived haplotypes than do the southernmost populations, as expected in recently originated populations [3]. It is possible to speculate that a factor beyond climatic conditions may have played a role in the post-glacial population dynamics of *Ae. albopictus.* Since approximately 15,000 years BP, human populations experienced a south-to-north expansion into East Asia [87]. Furthermore, several coastal settlements developed as a consequence of the post-glacial rise in sea level and the formation of coastal reef and lagoon/estuary systems, which allowed the exploitation of marine resources [88]. Finally, during the same period, in Southern China (at approximately 9,000 years BP), the transition from a hunting-gathering lifestyle to farming was initiated [89]. *Ae. albopictus,* unlike other tree-hole mosquitoes, is able to exploit human-associated habitats, so these human activities could have furnished the mosquito with an increased blood supply (humans themselves, along with their livestock) and suitable breeding sites; this could have favoured the inferred population expansion.

In this study, we focused on *Ae. albopictus* populations in the temperate regions of East Asia and in the Indochinese Peninsula. The results allow us to propose some hypotheses concerning the southernmost part of the range of the species, to be tested in future studies. SDM data showed the occurrence of climatically suitable areas across all of Sundaland. Therefore, it could be hypothesized that the emergence of Sundaland allowed population connectivity across the Indonesian islands and between these islands and mainland Asia. This scenario has recently been inferred for other mosquito species (such as *Anopheles vagus* s.l.) that can exploit open habitats [90] and for the fruit bat *Cynopterus brachyotis* [35]. Phylogeographic and historical demographic analyses based on extensive sampling throughout the Indonesian islands and New Guinea would allow us to test the possible occurrence of a similar scenario for *Ae. albopictus*. Our preliminary data show high genetic similarity in mitochondrial DNA among the populations from East Asia and 2 populations from Sulawesi Island (0.63%, mean sequence divergence) [91].

### Conclusions

Previous genetic studies on *Ae. albopictus* populations that were conducted using nuclear markers have shown a lack of genetic discontinuity across most of the geographic range of the species [41], [42]. Here, by integrating phylogeographic and SDM data, we have provided strong support for an evolutionary scenario in which this generalist mosquito persisted in widely interconnected populations in mainland East Asia during the last glacial phase. A similar scenario has been found in species with wide ecological flexibility in Australia [36], in Southeast Asia [35] and in the Western Palaearctic [34], [39], [37], [92], most likely as a result of their ability to exploit different habitats or to adapt to different resources or different hosts.

Historical demographic analyses also showed that *Ae. albopictus* experienced a demographic expansion that started in the last glacial phase and lasted until recently. The population expansion following the LGM, as expected, was driven by amelioration of climatic conditions at mid-latitudes and perhaps also by environmental changes attributable to human activities. On the contrary, the population expansion that occurred before the LGM (from 70,000 years BP) is an interesting exception. SDM, along with paleoenvironmental reconstructions and life traits of the species, suggest a positive role of Sundaland in the dynamics of *Ae. albopictus* populations. Also in this case, the ecological flexibility and adaptability of the species most likely allowed it to exploit the wide range of habitats offered by the emergence of Sundaland. Therefore, the case study of *Ae. albopictus* contributes to our understanding of East Asian phylogeography and supports the findings of those studies that showed the positive role of the glacial-induced emergence of lowlands in maintaining populations during glacial phases [36], [38], [86].

## Supporting Information

Figure S1 **Spatial analysis of molecular variance.** Fixation indices, obtained using the software SAMOVA, for the best clustering option for each pre-defined values of K. *F*_CT_: variation among groups of populations; *F*_SC_: variation among populations within groups; *F*_ST_: variation among populations among groups.
(TIF)
