# Supplementary figures and images for: Glacial History of a Modern Invader: Phylogeography and Species Distribution Modelling of the Asian Tiger Mosquito Aedes albopictus
Source: PLoS One. 2012 Sep 6;7(9):e44515. doi: 10.1371/journal.pone.0044515 (PMC3435282; doi:10.1371/journal.pone.0044515)

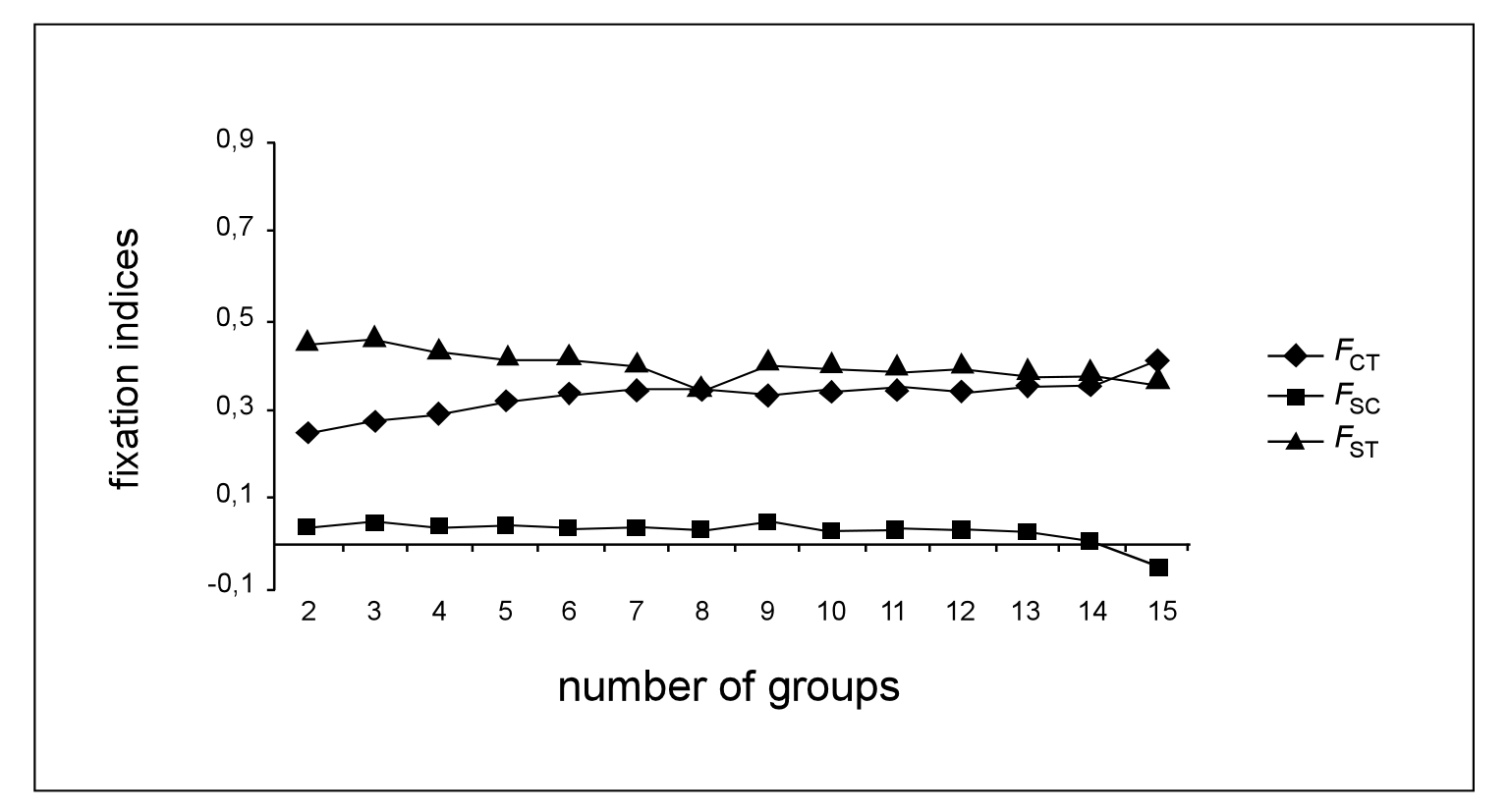

Supplement: Figure S1 — Spatial analysis of molecular variance. Fixation indices, obtained using the software SAMOVA, for the best clustering option for each pre-defined values of K. F CT: variation among groups of populations; F SC: variation among populations within groups; F ST: variation among populations among groups. (TIF) [file pone.0044515.s001.tif]
